# Caveolin-1 enhances brain metastasis of non-small cell lung cancer, potentially in association with the epithelial-mesenchymal transition marker SNAIL

**DOI:** 10.1186/s12935-019-0892-0

**Published:** 2019-06-28

**Authors:** Yeong-Jin Kim, Ju-Hwi Kim, Ok Kim, Eun-Jung Ahn, Se-Jeong Oh, Md Rashedunnabi Akanda, In-Jae Oh, Shin Jung, Kyung-Keun Kim, Jae-Hyuk Lee, Hyung-Seok Kim, Hangun Kim, Kyung-Hwa Lee, Kyung-Sub Moon

**Affiliations:** 1 Department of Neurosurgery, Chonnam National University Research Institute of Medical Science, Chonnam National University Hwasun Hospital and Medical School, 322 Seoyang-ro, Hwasun-eup, Hwasun-gun, Jeollanam-do 58128 South Korea; 2 Department of Pathology, Chonnam National University Research Institute of Medical Science, Chonnam National University Hwasun Hospital and Medical School, 322 Seoyang-ro, Hwasun-eup, Hwasun-gun, Jeollanam-do 58128 South Korea; 3 Lung and Esophageal Cancer Clinic, Chonnam National University Research Institute of Medical Science, Chonnam National University Hwasun Hospital and Medical School, Hwasun, Jeollanam-do South Korea; 4 Medical Research Center of Gene Regulation and Center for Creative Biomedical Scientists, Chonnam National University Medical School, Gwangju, South Korea; 5 Department of Forensic Medicine, Chonnam National University Medical School, 8 Hak-dong, Dong-gu, Gwangju, South Korea; 6 College of Pharmacy and Research Institute of Life and Pharmaceutical Sciences, Sunchon National University, Sunchon, Jeollanam-do South Korea; 7 Department of Pharmacology and Toxicology, Sylhet Agricultural University, Sylhet, 3100 Bangladesh

**Keywords:** Brain metastasis, Caveolin-1, Epithelial-mesenchymal transition, Non-small cell lung cancer, SNAIL

## Abstract

**Background:**

Caveolin-1 (Cav-1) plays an important role in the development of various human cancers. We investigated the relationship between Cav-1 expression and non-small cell lung cancer (NSCLC) progression in the context of brain metastasis (BM).

**Methods:**

Cav-1 expression was investigated in a series of 102 BM samples and 49 paired primary NSCLC samples, as well as 162 unpaired primary NSCLC samples with (63 cases) or without (99 cases) metastasis to distant organs. Human lung cancer cell lines were used for in vitro functional analysis.

**Results:**

High Cav-1 expression in tumor cells was observed in 52% (38/73) of squamous cell carcinomas (SQCs) and 33% (45/138) of non-SQCs. In SQC, high Cav-1 expression was increased after BM in both paired and unpaired samples of lung primary tumors and BM (53% vs. 84% in paired samples, *P *= 0.034; 52% vs. 78% in unpaired samples, *P *= 0.020). Although the difference in median overall survival in patients NSCLC was not statistically significant, high Cav-1 expression in tumor cells (*P *= 0.005, hazard ratio 1.715, 95% confidence index 1.175–2.502) was independent prognostic factors of overall survival on multivariate Cox regression analyses, in addition to the presence of BM and non-SQC type. In vitro assays revealed that Cav-1 knockdown inhibited the invasion and migration of lung cancer cells. Genetic modulation of Cav-1 was consistently associated with SNAIL up- and down-regulation. These findings were supported by increased SNAIL and Cav-1 expression in BM samples of SQC.

**Conclusions:**

Cav-1 plays an important role in the BM of NSCLC, especially in SQC. The mechanism may be linked to SNAIL regulation.

**Electronic supplementary material:**

The online version of this article (10.1186/s12935-019-0892-0) contains supplementary material, which is available to authorized users.

## Background

Lung cancer remains the 2nd common human cancer and the leading cause of cancer-associated death in the United States. The 5-year overall survival (OS) rate is only 19%, despite advanced treatment modalities [1]. Lung cancers are classified into several types based on the histologic classification. Non-small cell lung cancer (NSCLC), represented by adenocarcinoma (ADC) and squamous cell carcinoma (SQC), presents distinguished clinical courses with differences in treatment planning and prognosis prediction, compared to small cell lung cancer (SCLC) [2]. With the incidence of histologic subtype, ADC is the most common type, followed by SQC, and SCLC [3]. Brain metastasis (BM) develops in approximately 40% of patients with NSCLC and generally results in a dismal prognosis [4–6]. Effective therapy to manage the progressive biology of this disease continues to be debated. Therefore, a deeper understanding of the molecular pathogenesis underlying BM of NSCLC is essential for improving patient prognosis.

Caveolae, omega-shaped vesicular invaginations of the plasma membrane, consist of three major structural proteins: caveolin (Cav)-1, -2, and -3. Among them, Cav-1 is the substantial structural protein of caveolae. Caveolae are believed to function as vesicular transporters, cholesterol homeostasis modulators, and a signal platform where Cav-1 interacts with signaling molecules and regulates cell proliferation, differentiation, transformation, and metastasis [7–10]. An increasing number of studies have evaluated Cav-1 expression in cancer. Several studies have reported that Cav-1 plays a tumor-suppressive role, while others have revealed that increased expression of Cav-1 is implicated in tumor progression and metastasis [11–24].

Cav-1 functions as a tumor suppressor in SCLC and is inversely required for tumor cell survival and growth in NSCLC [25]. Cav-1 expression in pleomorphic carcinoma of the lung is correlated with a poor prognosis [26]. With the different expression of Cav-1 based on the histopathology, however, there are some debates in the clinical implication of Cav-1 in NSCLC patients [27–37]. Cav-1 exhibits increased expression during metastasis via interference with cell adhesion molecules, causing a loss of polarity during migration [38, 39]. Previous studies have reported that up-regulation of Cav-1 is related to epithelial-mesenchymal transition (EMT) and influences cancer cell motility [40, 41]. Reversely, EMT is associated with Cav-1 expression in human cancer [16, 42].

To date, few studies have reported that BM is correlated with Cav-1 expression in lung cancer [36, 37]. In the present study, Cav-1 expression was evaluated in relation to lung cancer histotypes, the presence of BM, and prognosis. Furthermore, a possible relationship with Cav-1 expression and EMT markers was investigated in NSCLC cell lines, along with changes in their migration and invasion abilities.

## Methods

### Human tissue specimens and clinical data

**Fig. 1:**
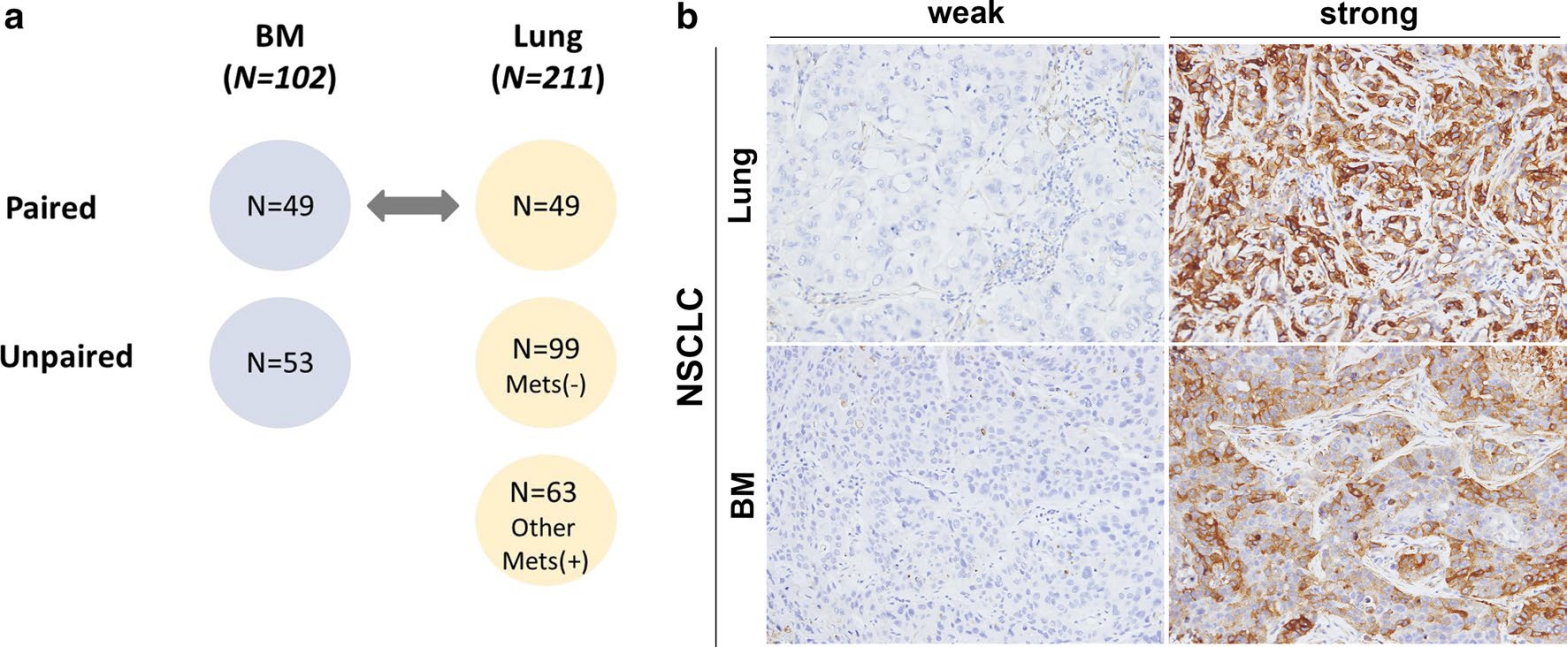
**a** Schematic classification of tissue specimens from the enrolled patients. A total of 102 brain metastasis (BM) samples consisting of 49 paired and 53 unpaired tissue specimens from non-small cell lung cancer (NSCLC) patients were evaluated. In 211 cases of primary NSCLC, there were 49 cases paired with BM, 99 cases without any systemic metastasis, and 63 cases with systemic metastases other than BM. **b** Representative images of Cav-1 immunohistochemical staining in cancer cells. Cav-1 expression varied from weak to strong in the tumor portion of primary NSCLC and BM

From January 1, 2007 to December 31, 2012, 105 cases affected by BM originating from NSCLC were enrolled (tissue specimens from the metastatic brain tumors of 102 patients were obtained by surgical resection; the remaining 3 patients were treated with Gamma knife radiosurgery for BM). A total of 211 NSCLC patients with primary cancer resected at the Lung and Esophageal Cancer Clinic were enrolled in this study. The patients were classified into four groups (Fig. 1a and Table 1). Group 1 comprised BM cases originating from NSCLC, Group 2 comprised primary NSCLC cases paired to the Group 1 cases, Group 3 comprised primary NSCLC cases without any systemic metastasis, and Group 4 comprised primary NSCLC cases with distant metastasis to organs other than the brain. Patients with preoperative radiotherapy or chemotherapy were excluded. Clinical information was retrospectively collected from clinical and pathological records. Pathological diagnosis was confirmed based on World Health Organization classifications. BM was confirmed by gadolinium-enhanced magnetic resonance imaging. OS was calculated as the time from the date of diagnosis to the date of death or the last follow-up visit. This study was approved by the Institutional Review Board of Chonnam National University Hwasun Hospital (CNUHH-2016-086). Written informed consent to use clinical data and pathological samples was obtained from patients or their legal surrogates.

**Table 1:** Number of cases used in this study according to sample groups

Variables	Group 1	Group 2	Group 3	Group 4
Age (mean 62.7 years)				
< 63	49	24	34	26
≥ 63	56	25	65	37
Sex				
Male	81	39	76	50
Female	24	10	23	13
Histological type				
SQC	27	19	29	25
ADC	62	24	56	31
LCC	16	6	14	7
Total	105^a^	49^b^	99	63

Group 1—Cases of BM from NSCLC

Group 2—Primary lung carcinoma cases paired to Group 1 cases

Group 3—Primary lung carcinoma cases without metastasis

Group 4—Primary lung carcinoma cases with distant metastasis to organs other than brain

SQC, squamous cell carcinoma; ADC, adenocarcinoma; LCC, large cell carcinoma

^a^Composed of 102 cases with open brain surgery and 3 cases with gamma knife radiosurgery (lung primary samples only)

^b^Not including 3 patients underwent gamma knife radiosurgery

### Immunohistochemistry (IHC)

All immunostained slides were evaluated twice by experienced pathologists (LJH and LKH) blinded to the clinical details. IHC was performed as described previously [43]. Tissue sections were immunostained with specific antibodies against Cav-1 (1:800, BD Biosciences, Franklin Lakes, NJ, USA) and SNAIL (1:100, Santa Cruz Biotechnology Inc., Dallas, TX, USA) using a Bond-Max autostainer system (Leica Microsystems, Buffalo Grove, IL, USA). Negative controls were processed in the absence of primary antibodies. The Cav-1 antibody stained the cytoplasmic borders and cytoplasm of cancer cells. The SNAIL antibody mainly stained the cytoplasm of cancer cells. The staining intensity in cancer cells was initially graded according to the following criteria: 0, no staining; 1, weak staining; 2, moderate staining; and 3, strong staining. Samples were grouped according to staining intensities of 0–1 (low expression) and 2–3 (high expression). These samples were also grouped according to staining intensity as described above (Fig. 1b).

### Cell culture and transfection

Human lung cancer cell lines (H1299, H157, H358, H520, H2170, and H1650) were obtained from the American Type Culture Collection (Manassas, VA, USA). Cells were cultured in Dulbecco’s Modified Eagle’s Medium (DMEM; WELGENE, Gyeongsan, South Korea) supplemented with 10% fetal bovine serum (FBS; WELGENE) in a humidified atmosphere of 5% CO_2_ at 37 °C. *Cav*-*1*-specific small interfering RNA (*si*RNA) (BIONEER, Daejeon, South Korea) was transfected into lung cancer cells using Lipofectamine™ 2000 (Invitrogen, Carlsbad, CA, USA) according to the manufacturer’s protocol. Control *si*RNA (BIONEER) was used as a negative control. *Cav*-*1* knockdown was confirmed at the mRNA and protein levels. *SNAIL*-specific *si*RNA (BIONEER) was transfected into Cav-1-overexpressing H157 cells, as above mentioned.

Knockdown and overexpression of *Cav*-*1* were conducted as described previously [44]. Recombinant lentivirus was purchased from Macrogen LentiVector Institute (Seoul, South Korea). pLKO.1 (Sigma–Aldrich, St. Louis, MO, USA) was used for small hairpin RNA (*sh*RNA) knockdown of *Cav*-*1*, and pWPI (Addgene, Cambridge, MA, USA) was used for overexpression of *Cav*-*1*. Supernatant with viral particles was obtained 48 h after incubation, passed through a 0.45-mm membrane filter (Nalgene, Rochester, NY, USA), and stored at − 70 °C until use. Transfection efficiency was assessed by fluorescence imaging and immunoblotting. A non-specific plasmid encoding β-galactosidase was used as a control in each transfection.

### Reverse transcriptase polymerase chain reaction (RT-PCR) and quantitative RT-PCR (qRT-PCR)

RT-PCR and qRT-PCR were performed as described previously [43]. The following primers were used: *GAPDH* (forward:5′-AGT TGT CAT GGA TGA CCT TGG C-3′; reverse:5′-ATC ACC ATC TTC CAG GAG CGA-3′), *SNAIL* (forward:5′-TCG GAA GCC TAA CTA CAG CGA-3′; reverse:5′-AGA TGA GCA TTG GCA GCG AG-3′), *Cav*-*1* (forward:5′-TTC GCC ATT CTC TCT TTC CT-3′; reverse:5′-CAG CTT CAA AGA GTG GGT CA-3′). Total RNA from cells was prepared using TRIzol reagent (Takara, Mountain View, CA, USA). After estimating the RNA concentration on the NanoDrop ND 1000 spectrophotometer (Thermo Fisher Scientific, Wilmington, DE, USA), 100 μg RNA were transcribed into cDNA using the LeGene Express 1st Strand cDNA Synthesis System Kit (LeGene Biosciences, San Diego, CA, USA). cDNA was amplified by h-Taq DNA Polymerase (SolGent, Daejeon, South Korea) and target primers. Amplification conditions were as follows: 15 min of denaturation at 95 °C, followed by 35 cycles of denaturation for 30 s at 95 °C, annealing for 30 s at 60 °C, and extension for 30 s at 72 °C, followed by a final extension for 7 min at 72 °C. No more than 35 PCR cycles were performed. Amplification of the endogenous reference gene GAPDH was used as an internal control. PCR products were electrophoresed on an agarose gel containing ethidium bromide and visualized using the Gel Doc EZ imager (Bio-Rad Laboratories, Hercules, CA, USA).

qRT-PCR was conducted using the veriQuest SYBR Green qPCR Kit (Affymetrix Inc., Santa Clara, CA, USA) and CFX96 Touch™ Real-time PCR Detection System (Bio-Rad Laboratories) running CFX manager software (Bio-Rad Laboratories). Amplification conditions were as follows: hot start for 10 min at 95 °C, followed by 40 cycles of denaturation for 15 s at 95 °C, annealing for 30 s at 60 °C, and elongation for 30 s at 72 °C. Gene expression was normalized relative to GAPDH expression in the same sample using the 2^−ΔΔCt^ method. All PCR experiments were repeated at least three times independently, and the average was calculated.

### Western blot analysis

The primary and secondary antibodies used in this study are listed in Additional file 1 (Table S1). Proteins were extracted from lysed cells using RIPA buffer (Bio Solution, Seoul, South Korea) supplemented with a protease inhibitor cocktail (Roche, Mannheim, Germany). After centrifugation, proteins (20–40 μg) were separated by 10% polyacrylamide gel electrophoresis containing 0.1% sodium dodecyl sulfate and electrophoretically transferred to polyvinylidene fluoride membranes (GE Healthcare Life Sciences, Marlborough, MA, USA). The membranes were probed with specific primary antibodies overnight, followed by anti-rabbit or anti-mouse immunoglobulin secondary antibodies conjugated to horseradish peroxidase. Bands were detected by an electrochemiluminescence system (Millipore, Burlington, MA, USA) and quantitated on the LAS-4000 luminescent image analyzer (Fujifilm, Tokyo, Japan).

### Cell invasion and migration assay

Cell invasion was measured using Transwells with chambers separated by filters with an 8 μm pore size (Corning Inc., Corning, NY, USA). Control or knockdown cells (1 × 10^5^ H157 cells and 1 × 10^5^ H1299 cells in 0.35 ml serum-free DMEM) were loaded into the upper chamber. For rescue assay by *SNAIL*-specific *si*RNA, 2 × 10^4^ mock or *Cav*-*1*-overexpressing H157 cells were loaded. Subsequently, the lower chamber was filled with 0.6 ml DMEM supplemented with 0.2% (w/v) bovine serum albumin and 5 μg/ml human plasma fibronectin (Calbiochem, San Diego, CA, USA). After incubation for 24 h at 37 °C, cells that had invaded the bottom side of the Transwell were fixed and stained using the Hemacolor Rapid Staining Kit (Merck, Darmstadt, Germany). Cell numbers from three to five random microscopic fields (each 0.5 mm^2^) were counted under a light microscope.

Gene-modulated H1299 and H157 cells in pairs were seeded into 96-well transparent tissue culture plates (ESSEN Bioscience, Ann Arbor, MI, USA) and cultured to 90% confluence in DMEM supplemented with 10% FBS for 24 h. Using a 96-well wound-maker (ESSEN Bioscience), a straight, uniform scratch was made through adherent cells. After the addition of serum-free DMEM, the plates were placed in IncuCyte™ (ESSEN Bioscience) and scanned at 0, 6, 12, and 24 h. The distance was measured using ZOOM software (ESSEN Bioscience) and normalized to 100%. Each experiment was conducted at least three times independently.

### Statistical analysis

All data were analyzed using SPSS version 23.0 software for Windows (Armonk, NY, USA). To analyze the effects of Cav-1 expression on categorical variables, Pearson Chi square test or Wilcoxon signed ranks test was used where appropriate. The effects of individual variables on OS were determined by univariate (Kaplan–Meier method with comparison by log-rank tests) and multivariate (Cox’s proportional hazards model) analyses. We analyzed the in vitro data using GraphPad Prism version 6.07 software for Windows (GraphPad, La Jolla, CA, USA). Data are presented as the mean ± standard error of the mean. The level of significance was set at *P *< 0.05.

## Results

### Cav-1 expression is related to BM in NSCLC

A total of 211 tissue samples obtained from primary NSCLC were tabulated by age, sex, histology, metastatic lesion, and Cav-1 expression (Table 2). Regarding histology, tumor cells of ADC showed lower Cav-1 expression (high Cav-1 expression, 28%), whereas SQC and large cell carcinoma showed relatively higher levels of Cav-1 expression (52% and 59%, respectively). Compared with the SQC and non-SQC groups, the differences in high Cav-1 expression were statistically significant (52% vs. 33%, respectively, *P *= 0.006).

**Table 2:** Cav-1 expression in 211 samples of primary NSCLC according to clininopathological variables

Variables	No.	Tumor Cav-1 expression		*P* value^a^
		Low	High	
Age				
< 63	84	53 (63%)	31 (37%)	0.556
≥ 63	127	75 (59%)	52 (41%)	
Sex				
Male	165	97 (59%)	68 (41%)	0.291
Female	46	31 (67%)	15 (33%)	
Histology				
SQC	73	35 (48%)	38 (52%)	0.006
Non-SQC	138	93 (67%)	45 (33%)	
Metastasis				
Absent	99	57 (58%)	42 (42%)	0.340
Brain	49	30 (61%)	19 (39%)	
Others	63	41 (65%)	22 (35%)	

SQC, squamous cell carcinoma

^a^Pearson Chi square test

The comparison of Cav-1 expression in 49 paired cases (Group 2: BM and primary lung cancer from the same patient) according to histology (SQC vs. non-SQC) revealed histotype-associated expression results (Table 3). In SQC, Cav-1 expression was significantly different between the primary lung and metastatic brain lesions. Primary lung lesions showed an intermediate level of Cav-1 expression (high Cav-1 expression, 10/19 [53%]), whereas BM showed a high level of Cav-1 expression (16/19, 84%, *P *= 0.034). This difference was due to the shift to high Cav-1 expression with BM development compared with the primary lung lesions. In non-SQC, tumor cells maintained a low level of Cav-1 expression in BM compared with the primary lesion (27% vs. 30%, respectively, *P *= 0.564).

**Table 3:** Comparison of tumor Cav-1 expression in 49 paired cases of lung primary and BM according to histology

	BM			*P* value^b^
	Low	High	High (%)^a^	
Lung primary				
SQC				
Low	2	7	16/19 (84%)	0.034
High	1	9		
High (%)^a^	10/19 (53%)			
Non-SQC				
Low	20	1	8/30 (27%)	0.564
High	2	7		
High (%)^a^	9/30 (30%)			

SQC, squamous cell carcinoma

^a^Represented as proportion of cases with high Cav-1 expression

^b^Wilcoxon signed ranks test

The analysis of Cav-1 expression between all BM (*N *= 102) and primary NSCLC (*N *= 211) cases revealed a similar pattern (Table 4). In SQC, Cav-1 expression was frequently higher in BM than the primary lung lesion (cases with high Cav-1 expression, 78% vs. 52%, respectively, *P *= 0.020). In non-SQC, tumor cells showed low Cav-1 expression in both primary lung cancer and BM (cases with high Cav-1 expression, 33% vs. 27%, respectively, *P *= 0.368).

**Table 4:** Comparison of Tumor Cav-1 expression in 102 samples of BM and 211 samples of primary NSCLC according to histology

	No.	Tumor Cav-1		*P* value^a^
		Low	High	
SQC				
BM	27	6 (22%)	21 (78%)	0.020
Lung	73	35 (48%)	38 (52%)	
Sum	100	41 (41%)	59 (59%)	
Non-SQC				
BM	75	55 (73%)	20 (27%)	0.368
Lung	138	93 (67%)	45 (33%)	
Sum	213	148 (70%)	65 (30%)	
Total				
BM	102	61 (60%)	41 (40%)	0.884
Lung	211	128 (61%)	86 (39%)	
Sum	313	189 (60%)	127 (40%)	

SQC, squamous cell carcinoma

^a^Pearson Chi square test

Collectively, tumor cells in the primary lung lesion showed higher Cav-1 expression in the SQC group than in the non-SQC group. Cav-1 expression in the SQC group was further elevated in tumor cells of BM compared to the primary lung lesions.

### Cav-1 expression in the primary lung lesion may relate to survival in NSCLC patients

**Fig. 2:**
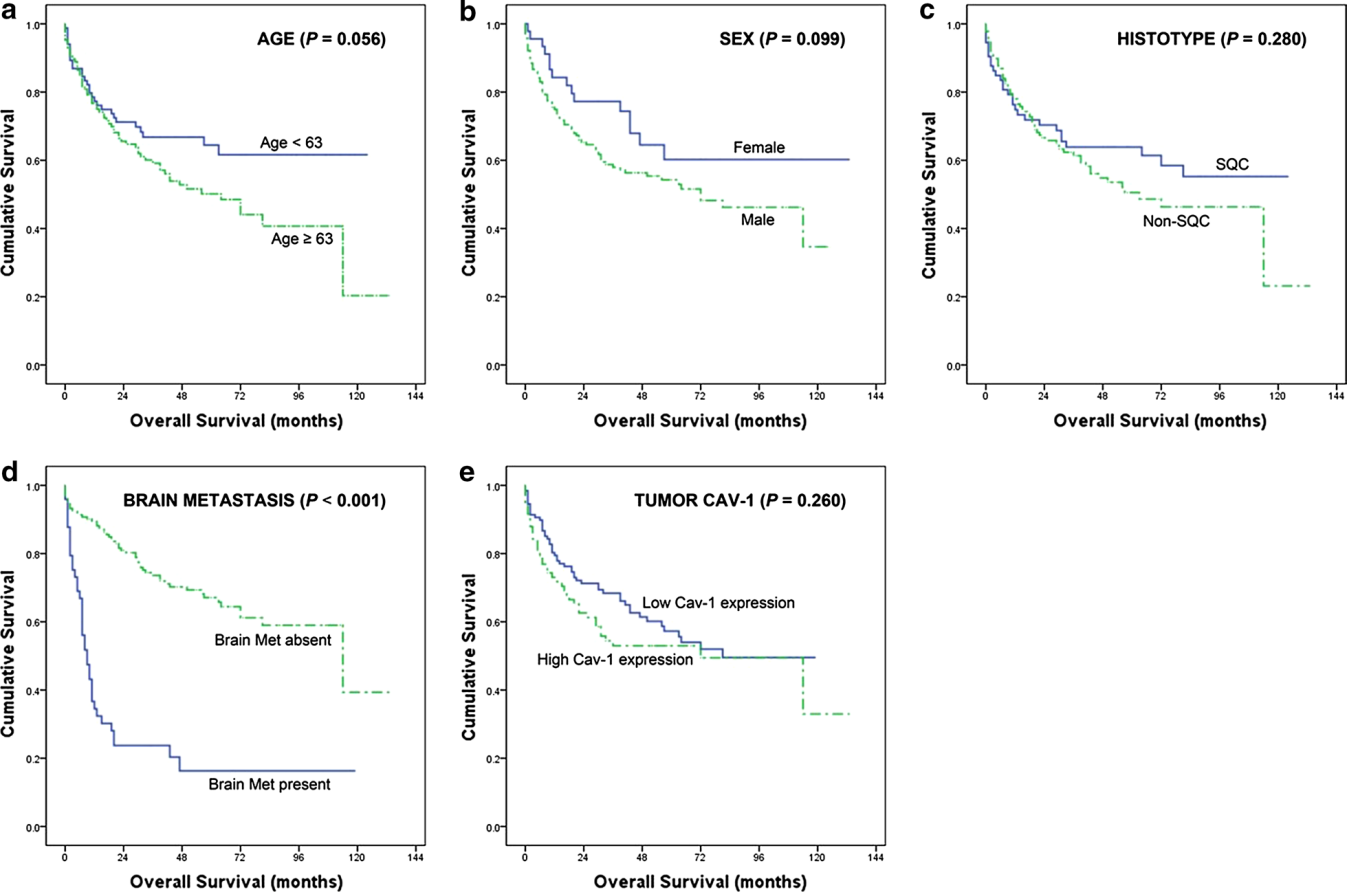
Kaplan–Meier analyses of overall survival in 211 enrolled NSCLC patients according to different clinicopathological factors of primary lung cancer (overall comparison was estimated using a log-rank test). **a** Age. **b** Sex. **c** Histotype of primary cancer. **d** Presence of BM. **e** Intensity of tumor Cav-1 expression

Clinical features, including age, sex, histology, presence of BM, and Cav-1 expression in the tumor cells, of 211 primary NSCLC patients were analyzed (Table 5 and Fig. 2). Although the median OS was shorter in patients with high Cav-1 expression than in those with low Cav-1 expression in tumor cells, the difference was not statistically significant (71.0 vs. 73.1 months, respectively, *P *= 0.260). Univariate analyses revealed that the presence of BM was significantly prognostic of OS (*P *< 0.001). Moreover, multivariate Cox regression analyses revealed that the presence of BM (*P *< 0.001, hazard ratio [HR] 0.181, 95% confidence index [CI] 0.124–0.264), non-SQC type (*P *= 0.007, HR 1.762, 95% CI 1.168–2.659), and Cav-1 expression in tumor cells (*P *= 0.005, HR 1.715, 95% CI 1.175–2.502) were independent prognostic factors of OS. Interestingly, Cav-1 expression in BM was partly related with OS in NSCLC patients (*P *= 0.116, Additional file 2: Figure S1).

**Table 5:** Univariate and multivariate analysis for overall survival predictors in 211 patients with primary NSCLC

Characteristics	No.	Mean survival (months)	*P* value (univariate)^a^	*P*-value (multivariate)^a^	Hazard ratio	95% Confidence index
Age						
< 63	84	83.1	0.056	0.109	1	0.935–1.942
≥ 63	127	66.3			1.348	
Sex						
Male	165	68.7	0.099	0.162	1	0.456–1.141
Female	46	90.5			0.721	
Histology						
SQC	73	78.7	0.280	0.007	1	1.168–2.659
Non-SQC	138	69.9			1.762	
BM						
Absent	162	87.7	< 0.001	< 0.001	0.181	0.124–0.264
Present	49	28.3			1	
Tumor Cav-1						
Low	128	73.1	0.260	0.005	1	1.175–2.502
High	83	71.0			1.715	

SQC, squamous cell carcinoma

^a^Cox proportional hazards model for multivariate analysis

### Knockdown of Cav-1 decreases cell migration and invasion

**Fig. 3:**
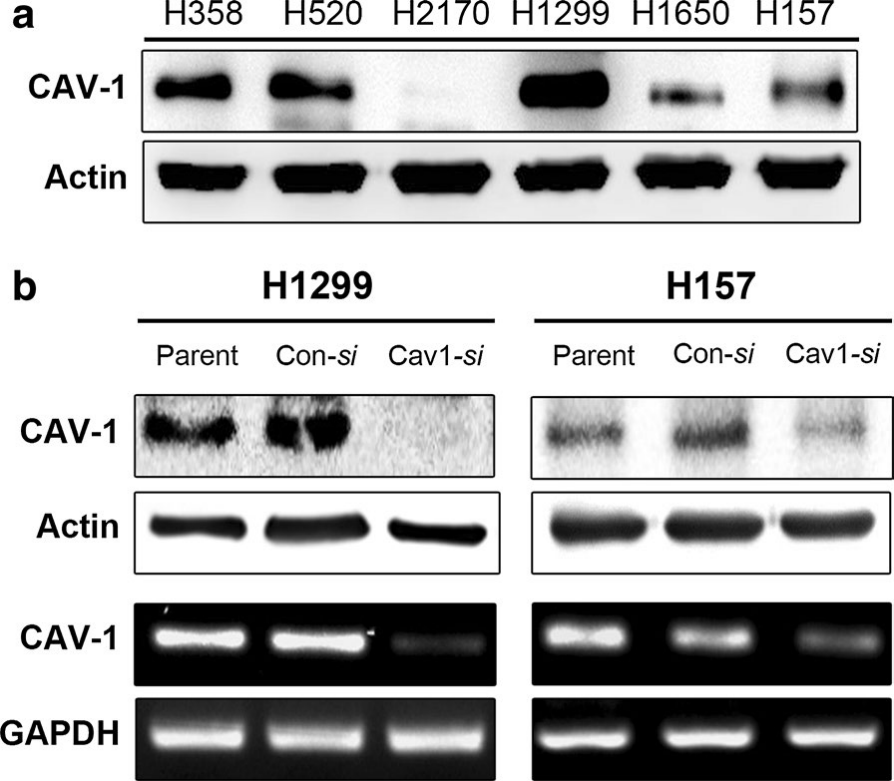
Cav-1 expression in human lung cancer cell lines. **a**Western blot analysis showing baseline expression of Cav-1 in various lung cancer cell lines. **b** Western blot and RT-PCR analysis of Cav-1 protein and mRNA expression in H1299 and H157 cells, respectively, after transient knockdown using *si*RNA

**Fig. 4:**
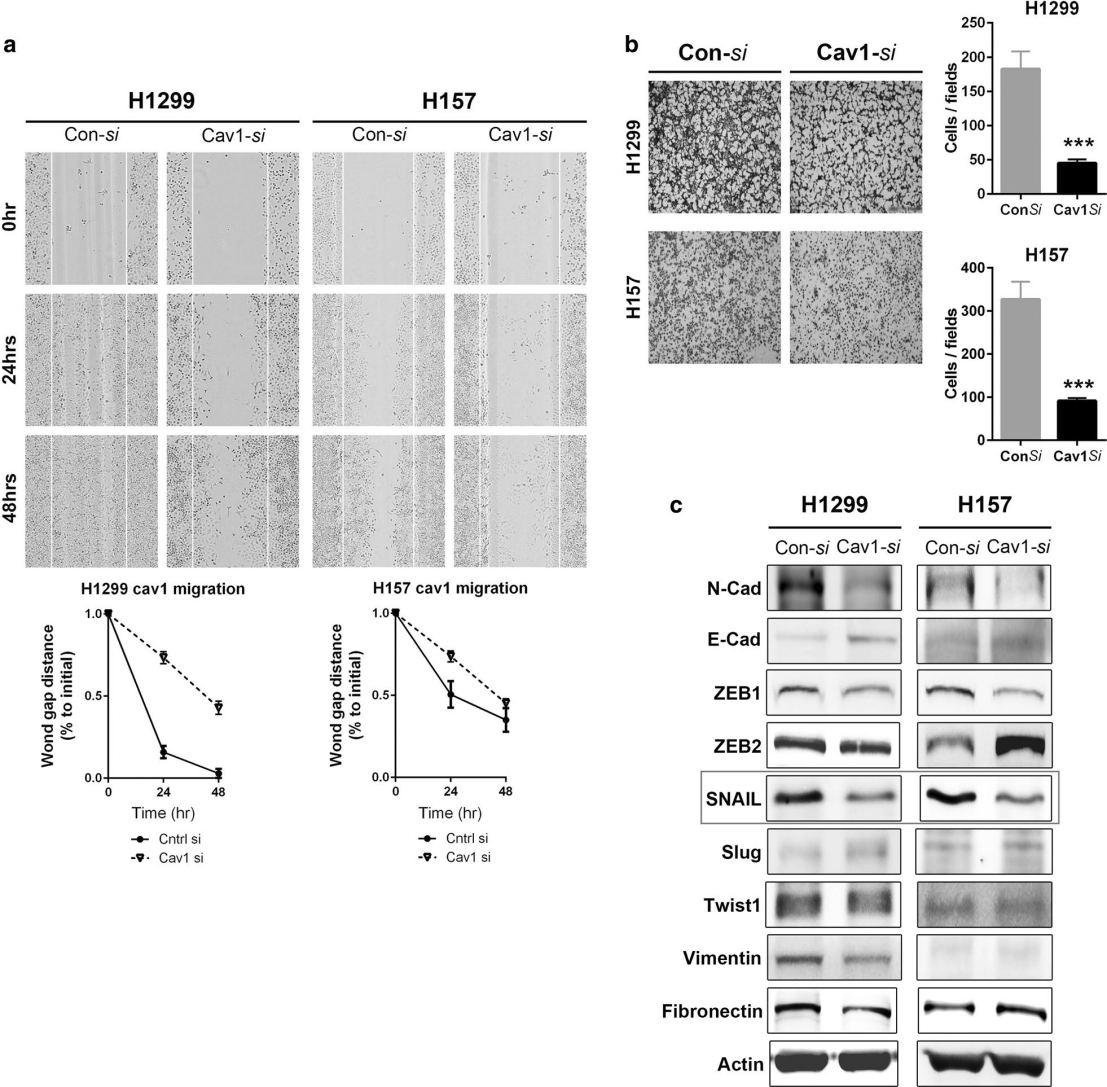
**a** Effect of Cav-1 knockdown on the migration of human SQC cell lines. Cav-1 knockdown significantly decreased H1299 and H157 cell invasion, as shown by wound gap distance. **b** Effect of Cav-1 knockdown on the invasion of human SQC cell lines. Cav-1 knockdown significantly decreased H1299 and H157 cell invasion, as shown by cell counts per field. Bar graphs show the mean ± standard error of the mean (***P < 0.001). **c** Altered expression of EMT markers and regulators by Cav-1 knockdown in both cell lines. Cav-1 knockdown reduced the expression of EMT markers, including N-cadherin and fibronectin, and reversely increased the expression of E-cadherin, representative epithelial marker. Among the EMT-regulating genes, SNAIL was consistently reduced by Cav-1 knockdown in both cell lines

To investigate the role of Cav-1 in lung cancer cells, Cav-1 expression was evaluated by Western blotting and RT-PCR in various lung cancer cell lines. Among them, H157 and H1299 cell lines with up-regulated expression of Cav-1 were selected. Knockdown of *Cav*-*1* using *si*RNA suppressed endogenous *Cav*-*1* mRNA and protein expression in these cell lines (Fig. 3). *Cav*-*1* knockdown in lung cancer cell lines reduced cell migration in vitro (Fig. 4a). After 24 h, the artificial wound gaps became significantly narrower in plates of mock-transfected than *Cav*-*1 si*RNA-transfected H1299 and H157 cells (wound gap distance decrease, 68% and 27%, respectively). *Cav*-*1* knockdown in lung SQC cell lines significantly reduced cell invasion in vitro, i.e., a significantly smaller number of H1299 and H157 cells transiently transfected with *Cav*-*1 si*RNA migrated through the membrane compared with mock-transfected cells (cell number decrease, 75% and 73%, respectively) (Fig. 4b).

### Knockdown of Cav-1 decreases expression of EMT markers and EMT-regulating genes

To identify factors associated with alterations in cell invasion and migration caused by modulation of Cav-1 expression, the expression levels of EMT markers and EMT-regulating genes were determined in H1299 and H157 cells (Fig. 4c). Transient *Cav*-*1* knockdown in both lung cancer cell lines reduced the expression of EMT markers, including N-cadherin and fibronectin, at the protein and mRNA levels. Among the EMT-regulating genes, SNAIL was consistently down-regulated by *Cav*-*1* knockdown in both cell lines.

### Increased Cav-1 expression is accompanied by increased SNAIL expression in SQC

**Fig. 5:**
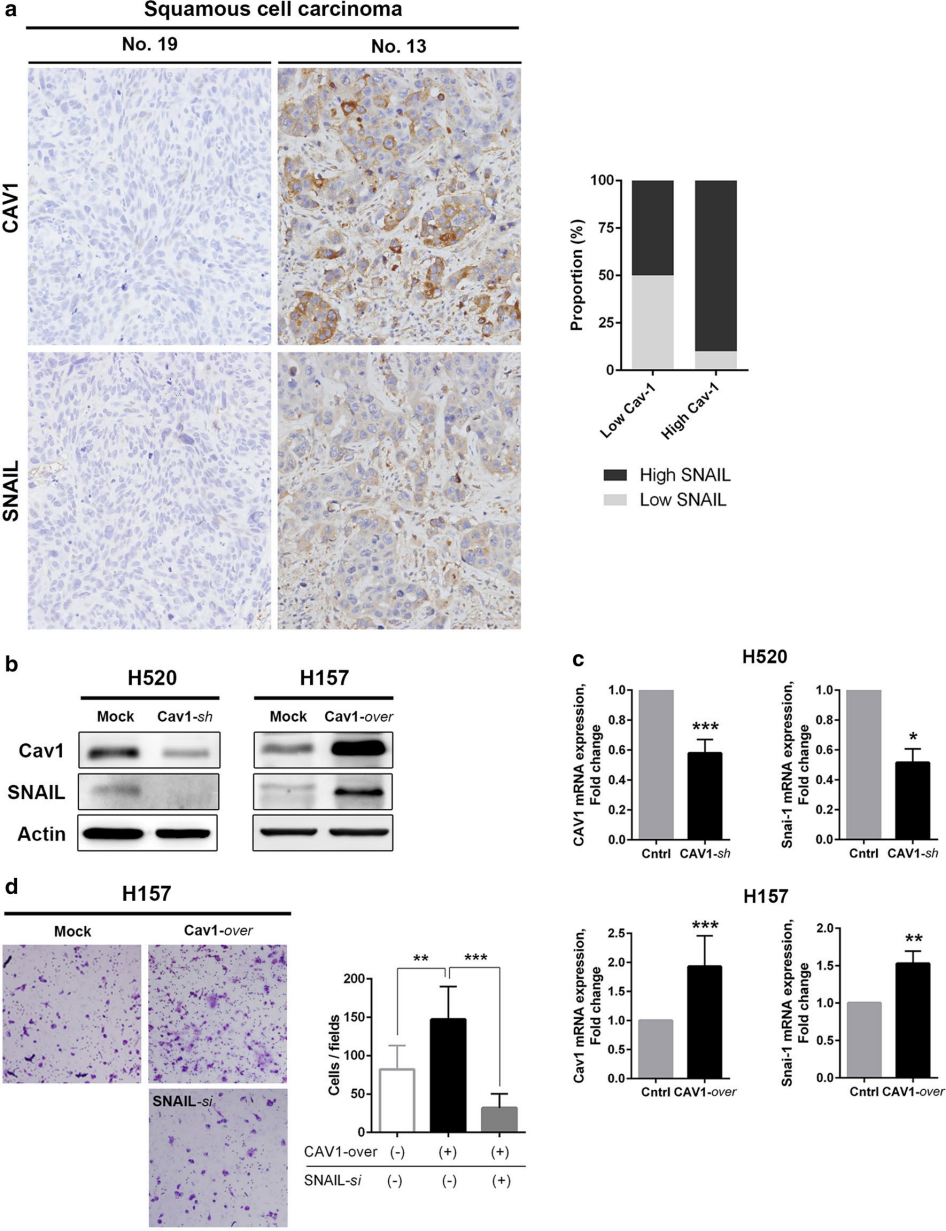
**a** Representative images of immunohistochemical staining for Cav-1 and SNAIL in BM of the SQC type. Note that the pattern of Cav-1 and SNAIL expression showed a similar direction in the SQC group. The intensity of SNAIL expression increased in proportion to Cav-1 expression in the SQC type (high SNAIL expression: 50% in low intensity of Cav-1 vs. 90% in high intensity of Cav-1, *P *= 0.051). **b**, **c** Genetic modulation of Cav-1 in lung SQC cell lines (H520 and H157). *sh*RNA knockdown of Cav-1 in H520 cells (Cav-1-*sh*) and overexpression of Cav-1 in H157 cells (Cav-1-*over*) led to changes in SNAIL expression in the same direction at both the protein (**b**) and mRNA (**c**) levels. **d** Rescue assay for possible link between Cav-1 and SNAIL. Increased invasion ability was observed in *Cav*-*1*-*over* H157 cells compared to mock cells. This ability was reduced by SNAIL-specific *si*RNA for *Cav*-*1*-*over* H157 cells (**d**) (*P < 0.05, **P < 0.005, ***P < 0.001)

Based on the IHC analysis of Cav-1 expression, Cav-1 expression was higher in BM than in primary lung cancer of the SQC type. In vitro assays revealed that *Cav*-*1* knockdown led to decreased invasion and migration abilities with a reduction in SNAIL expression. To verify the relationship between Cav-1 and SNAIL in tissue samples, we compared SNAIL expression with Cav-1 expression according to histologic type. In the SQC type, the intensity of SNAIL expression increased concordantly with Cav-1 expression in BM samples (Fig. 5a). In contrast, SNAIL expression was inversely related to Cav-1 expression in the ADC group (Additional file 3: Figure S2).

In lung SQC cell lines, genetic modulation of *Cav*-*1* was accompanied by SNAIL expression (Fig. 5b, c). Knockdown of *Cav*-*1* by *sh*RNA in H520 cells led to decreased expression of SNAIL at both the protein and mRNA levels. In contrast, overexpression of *Cav*-*1* in H157 cells was related to increased expression of SNAIL. Dual immunofluorescent staining of Cav-1 and SNAIL performed on mock or *Cav*-*1*-overexpressing H157 cells supported this finding (Additional file 4: Figure S3). Co-localization of Cav-1 and SNAIL was enhanced in Cav-1-overexpressing cells. Increased invasion ability of *Cav*-*1*-overexpressing H157 cells was reduced by a rescue assay using *SNAIL*-specific *si*RNA, implying a functional link between Cav-1 and SNAIL (Fig. 5d).

## Discussion

The function of Cav-1 in tumors is still under debate. It is unclear whether Cav-1 inhibits or promotes tumor growth and progression. Different types of tumor cells are correlated with Cav-1 in various ways [11–24]. In lung cancer, contradictory roles of Cav-1 have been reported [25–37]. Based on an investigation of Cav-1 expression in distinct lung cancer histology, Cav-1 expression was decreased in 95% of SCLC cell lines but maintained in 76% of NSCLC cell lines [25]. In NSCLC, Cav-1 expression was higher in secondary lesions than in primary tumors, as the histology-related tumor aggressiveness increased [36]. Other studies have reported higher Cav-1 expression in pT1 than in pT2–pT4 tumor cells of ADC but lower Cav-1 expression in pT1–pT2 than in pT3–pT4 tumor cells of SQC [35].

In our 211 primary lung cancer cases, higher Cav-1 expression was seen in the SQC (52%) group than in the non-SQC group (33%), supporting a relationship between Cav-1 expression and lung cancer histology. In a comparison of Cav-1 expression between primary lung and secondary BM (53% vs. 84% in paired samples, 52% vs. 78% in whole samples), Cav-1 expression in tumor cells was higher in secondary BM than in primary lung lesions of SQC. This finding suggests that Cav-1 specifically affects BM in lung SQC. Previous investigations have reported the impact of Cav-1 on the tumor microenvironment in some cancers, such as prostate, breast, and pancreas [45–48]. In our study, we found that a low Cav-1 level in stromal cells was related to BM in NSCLC, especially in ADC (data not shown). Although these data should be verified in a larger case series and molecular studies, Cav-1 in the stromal component may be negatively correlated with that in BM of ADC. Recently, Cav-1 expression of fibroblast or macrophage in stromal component has been investigated in primary or secondary lung cancer [49–51].

In the aspect of survival, the poor survival affected by high Cav-1 expression was also observed in other systemic cancer [20–22, 24]. In lung cancer, overexpression of Cav-1 in primary cancer cells was significantly related to poor prognosis in patients with various histotypes [26–31, 34]. On the contrary, some investigations revealed that the survival of patients with NSCLC, especially in ADC, was positively correlated with Cav-1 expression [32, 33, 42]. Our results revealed that Cav-1 expression in primary NSCLC is associated with poor survival (*P *= 0.005, 1.715 HR, 1.175–2.502 95% CI), in addition to the presence of BM and non-SQC histology. Furthermore, Cav-1 expression in BM was associated with OS in NSCLC patients, although the difference was not significant (*P *= 0.116). A similar result was reported that high expression of Cav-1 in BM was correlated with a poor survival rate in lung cancer patients [37]. Although the clinical relevance of Cav-1 expression was not verified according to the histological classification, our study suggested that high Cav-1 expression in tumor cells is definitively related in BM of SQC type and can be a poor independent prognostic factor in NSCLC.

**Fig. 6:**
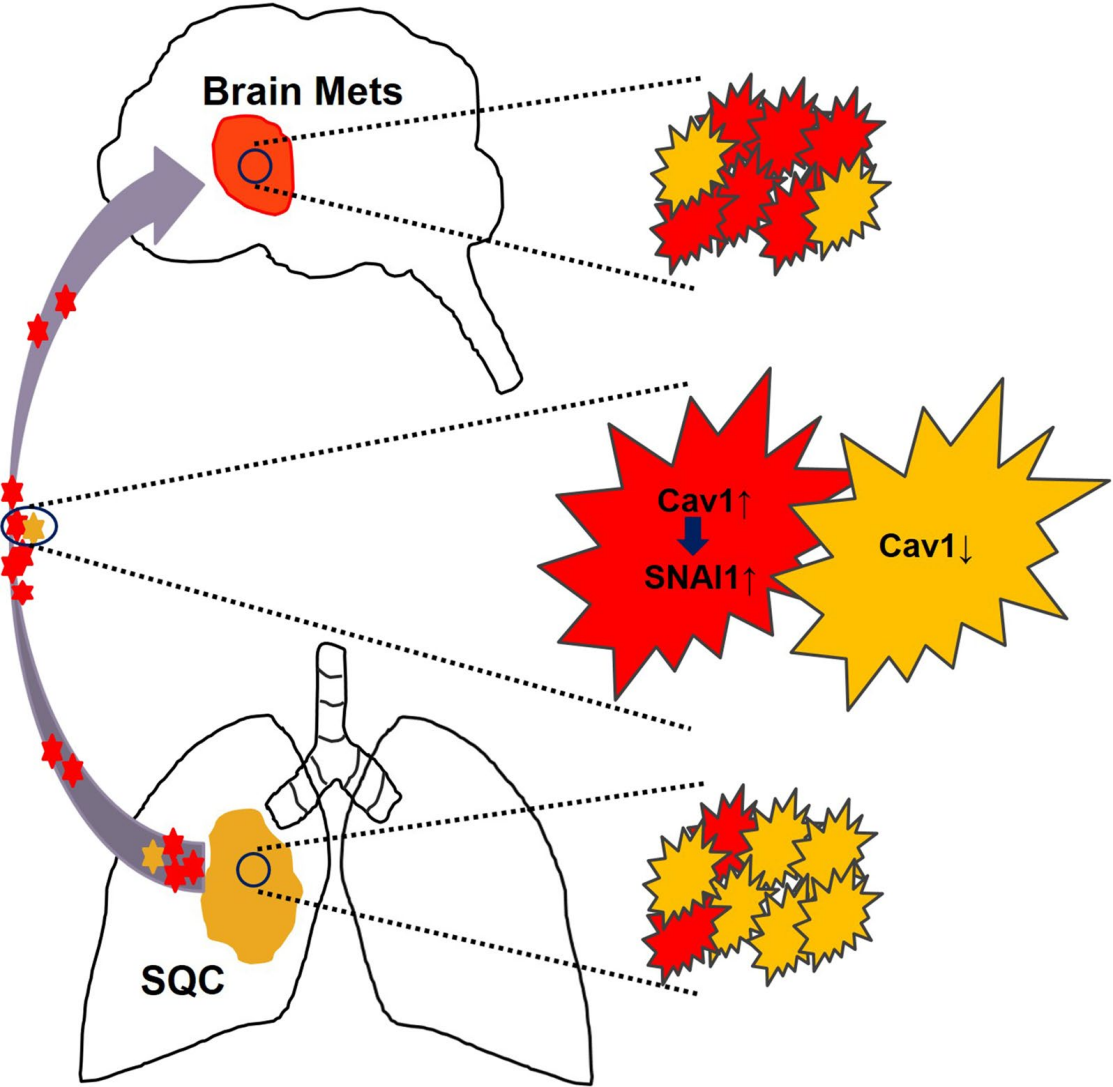
Scheme representing possible role of Cav-1 in BM of SQC lung cancer. High Cav-1 expressing SQC in primary lung cancer is associated increased SNAIL, EMT marker. These cells have more chance to metastasize to the brain compared to low Cav-1 expressed SQC cells

During BM from lung cancer, polarized epithelial cells transform from loose mesenchymal cells, which have enhanced migratory capacity, invasiveness, and production of extracellular matrix, via the EMT [52]. The prominent molecular change is the loss of E-cadherin, a key cell-to-cell adhesion molecule that suppresses tumor metastasis [53]. A number of transcription factors, such as SNAIL, SLUG, ZEB1, ZEB2, and TWIST, are well-known EMT inducers, representing increased EMT markers, such as N-cadherin, fibronectin, vimentin, and matrix metalloproteinase. SNAIL strongly represses E-cadherin expression as a potent transcription factor involved in the EMT [54, 55]. SNAIL is highly expressed in NSCLC, and the up-regulation of SNAIL is associated with poor prognosis by promoting tumor progression via the CXCR2 axis [56]. In addition, Cav-1 expression is increased during metastasis via interference with cell adhesion molecules and a loss of polarity in migration [38, 39]. It has also been reported that upregulation of Cav-1 is related to the EMT and influences cancer cell motility [40]. In pancreatic and gastric cancers, the possible association between Cav-1 and SNAIL has been verified, albeit in opposing ways [16, 41]. In the present study, Cav-1 knockdown in lung SQC cell lines resulted in decreased invasion and migration, possibly via a reduction in SNAIL expression. This relationship was supported by IHC data demonstrating that SNAIL expression was increased together with Cav-1 expression in BM samples of the SQC type. These results indicate that Cav-1 regulates cell motility and may affect the EMT via SNAIL, especially in BM of SQC lung cancer (Fig. 6).

## Conclusion

Cav-1 is believed to play an important role in BM of NSCLC depending on the histotypes. With the finding that Cav-1 expression is higher in BM than primary cancer of the SQC type, we provide pivotal insight into the possible role of Cav-1 on EMT via SNAIL. Clinical findings that high Cav-1 expression is associated with poor clinical outcomes suggest that elucidating Cav-1 function may present a novel and promising therapeutic approach for the treatment of NSCLC. Further studies should be investigated to reveal the connecting mechanism between Cav-1 and the EMT, as well as cancer progression in NSCLC.

## Additional files

**Additional file 1: Table S1.** Antibodies used for Western blot analysis.

**Additional file 2: Figure S1.** Kaplan–Meier analyses of OS for NSCLC patients according to the intensity of Cav-1 expression in BM (n = 105). NSCLC patients with high Cav-1 expression in BM had a shorter survival period than did those with low Cav-1 expression, although the difference was not significant (*P *= 0.116).

**Additional file 3: Figure S2.** Representative images of immunohistochemical staining for Cav-1 and SNAIL in BM of ADC. In contrast to SQC, the intensity of SNAIL expression was inversely related to Cav-1 intensity in ADC (high SNAIL expression: 94% in low intensity of Cav-1 vs. 67% in high intensity of Cav-1, *P *= 0.023).

**Additional file 4: Figure S3.** Immunofluorescence (IF) of Cav-1 and SNAIL in Cav-1-overexpressing H157 cells. IF was performed as described below: Mock and *Cav*-*1*-*over* H157 cells were grown on Lab-Tek II chamber slides (Thermo Fisher Scientific). Cells were fixed in 4% paraformaldehyde (Sigma-Aldrich) and permeabilized in 0.1% Tween-20 for 20 min each, and then blocked in 2% bovine serum albumin for 30 min. The cells were co-incubated overnight at 4  °C in mouse anti-Cav-1 (1:50, BD Biosciences, Franklin Lakes, NJ, USA, Catalog# BD 610407) and rabbit anti-SNAIL (1:50, Santa Cruz Biotechnology Inc., Dallas, TX, USA, Catalog # sc-28199) primary antibodies. After washing in PBS, the cells were co-incubated in goat anti-mouse IgG (1:100, Life Technologies, Catalog # A-11001) and goat anti-rabbit IgG (1:100, Life Technologies Catalog # A1011) secondary antibodies for 1 h at room temperature. Cells were then incubated with DAPI (1:1000) for 20 min followed by washing in PBS. The chamber slides were mounted with antifade mounting media and imaged in a confocal microscope (Olympus FV1000). The intensity of Cav-1 and SNAIL were determined by using Image J software. Increased expression of both Cav-1 and SNAIL was observed in Cav-1-over H157 cells compared to mock cells (**P < 0.005)

## Data Availability

The datasets used and analyzed during the current study are available from the corresponding author on reasonable request.

## Abbreviations

ADC: adenocarcinoma
BM: brain metastasis
Cav-1: caveolin-1
DMEM: Dulbecco’s Modified Eagle’s Medium
EMT: epithelial-mesenchymal transition
FBS: fetal bovine serum
NSCLC: non-small cell lung cancer
OS: overall survival
SCLC: small cell lung cancer
*sh*RNA: small hairpin RNA
*si*RNA: small interfering RNA
SQC: squamous cell carcinoma
