# Supplementary material for: Caveolin-1 enhances brain metastasis of non-small cell lung cancer, potentially in association with the epithelial-mesenchymal transition marker SNAIL
Source: Cancer Cell Int. 2019 Jun 28;19:171. doi: 10.1186/s12935-019-0892-0 (PMC6599320; doi:10.1186/s12935-019-0892-0)
Supplement: Supplementary file 1 — Additional file 1: Table S1. Antibodies used for Western blot analysis. [file 12935_2019_892_MOESM1_ESM.docx]

| Table S1. Antibodies used for Western blot analysis. | | | | | |
| --- | --- | --- | --- | --- | --- |
| Antibody | Molecular weight (kDa) | Dilution | Secondary antibody | Manufacturer | Catalogue No. |
| Caveolin-1 | 24 kDa | 1:1000 | mouse | BD | BD-610407 |
| N-cadherin | 100 kDa | 1:1000 | rabbit | Abcam | ab12221 |
| E-cadherin | 120 kDa | 1:100 | rabbit | DAKO | M3612 |
| ZEB1 | 125 kDa | 1:500 | rabbit | Bethyl | IHC-00419 |
| ZEB2 | 157 kDa | 1:500 | rabbit | Sigma | HPA003456 |
| SNAIL | 29 kDa | 1:1000 | rabbit | Santa Cruz | sc-28199 |
| SLUG | 30 kDa | 1:500 | rabbit | Abcam | ab38551 |
| TWIST | 21 kDa | 1:1000 | mouse | Abcam | ab50887 |
| Vimentin | 57 kDa | 1:1000 | rabbit | Abcam | Ab8978 |
| Fibronectin | 230-250 kDa | 1:1000 | mouse | Abcam | Ab6328 |
| Actin | 42 kDa | 1:10000 | mouse | BD | BD-612656 |
