# Supplementary figures and images for: Caveolin-1 enhances brain metastasis of non-small cell lung cancer, potentially in association with the epithelial-mesenchymal transition marker SNAIL
Source: Cancer Cell Int. 2019 Jun 28;19:171. doi: 10.1186/s12935-019-0892-0 (PMC6599320; doi:10.1186/s12935-019-0892-0)

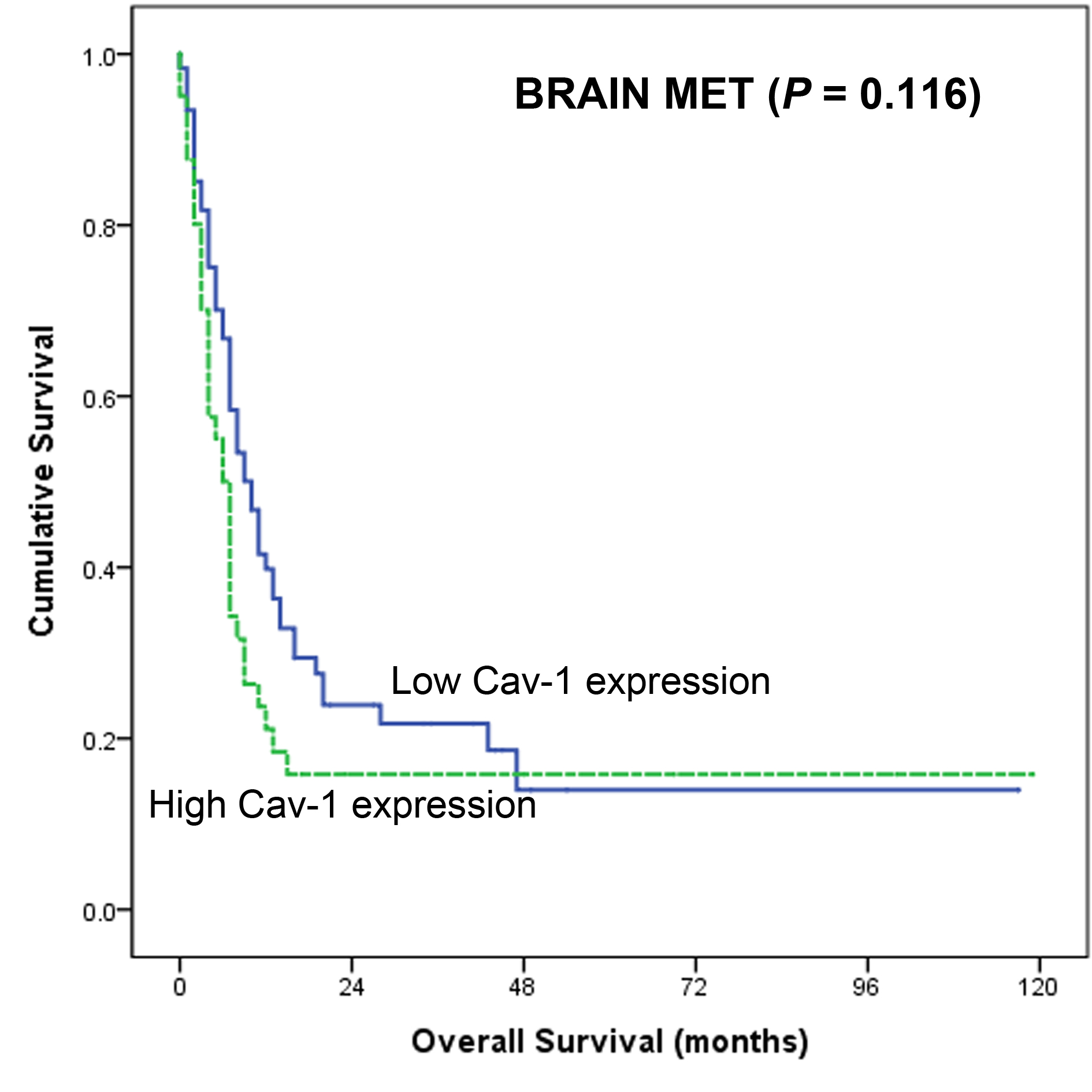

Supplement: Supplementary file 2 — Additional file 2: Figure S1. Kaplan–Meier analyses of OS for NSCLC patients according to the intensity of Cav-1 expression in BM (n = 105). NSCLC patients with high Cav-1 expression in BM had a shorter survival period than did those with low Cav-1 expression, although the difference was not significant (P = 0.116). [file 12935_2019_892_MOESM2_ESM.jpg]

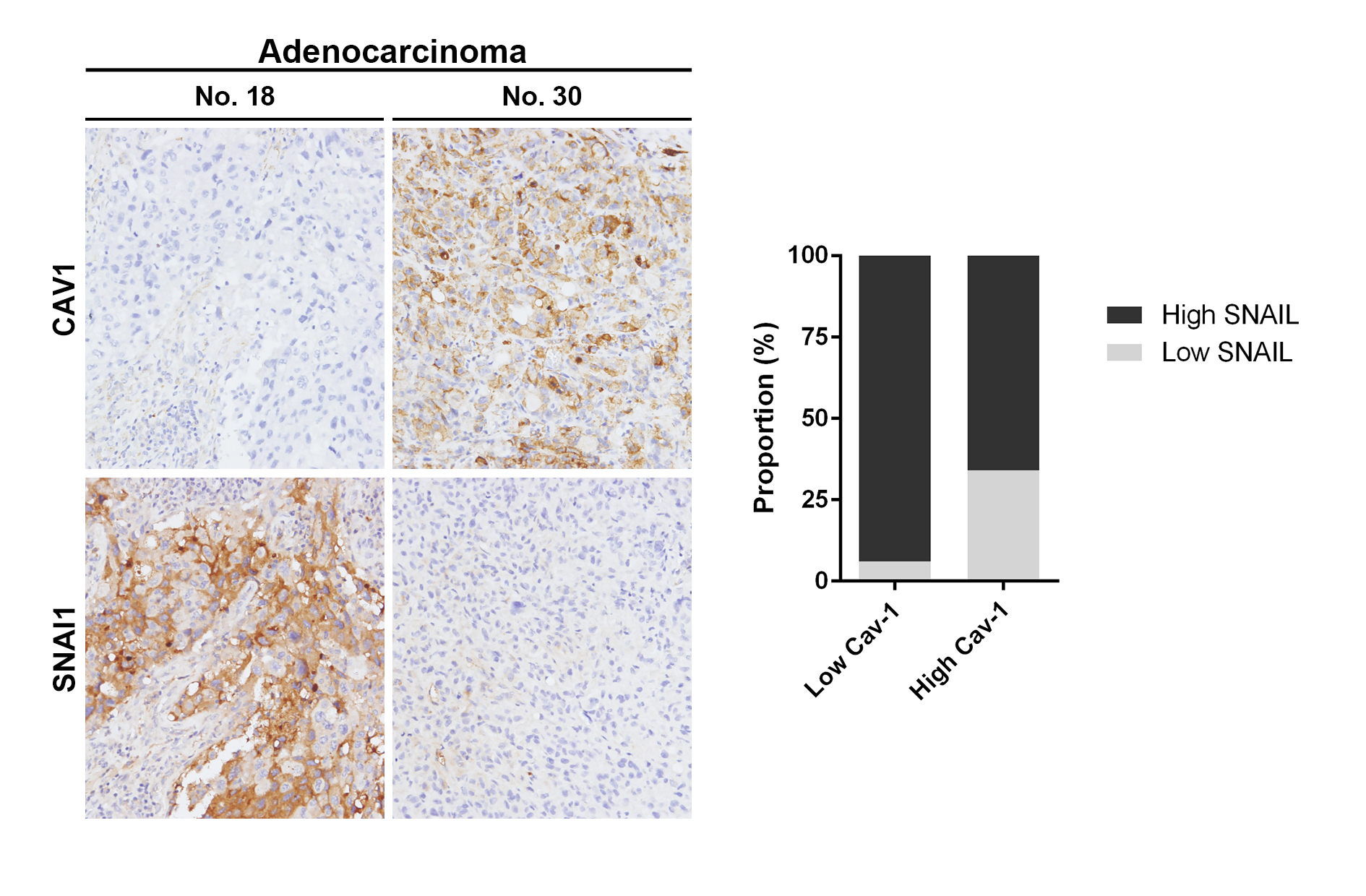

Supplement: Supplementary file 3 — Additional file 3: Figure S2. Representative images of immunohistochemical staining for Cav-1 and SNAIL in BM of ADC. In contrast to SQC, the intensity of SNAIL expression was inversely related to Cav-1 intensity in ADC (high SNAIL expression: 94% in low intensity of Cav-1 vs. 67% in high intensity of Cav-1, P = 0.023). [file 12935_2019_892_MOESM3_ESM.jpg]

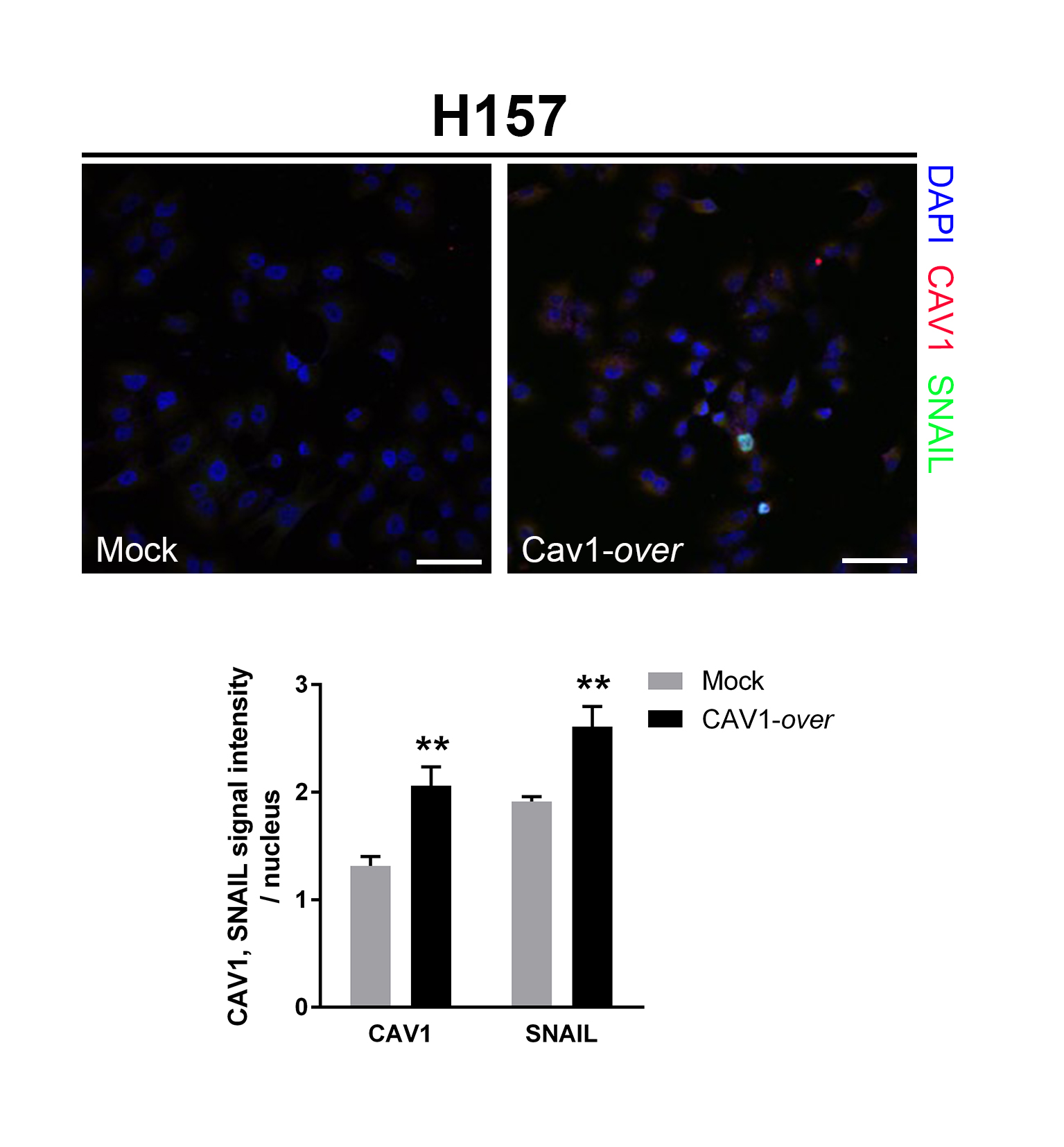

Supplement: Supplementary file 4 — Additional file 4: Figure S3. Immunofluorescence (IF) of Cav-1 and SNAIL in Cav-1-overexpressing H157 cells. IF was performed as described below: Mock and Cav-1-over H157 cells were grown on Lab-Tek II chamber slides (Thermo Fisher Scientific). Cells were fixed in 4% paraformaldehyde (Sigma-Aldrich) and permeabilized in 0.1% Tween-20 for 20 min each, and then blocked in 2% bovine serum albumin for 30 min. The cells were co-incubated overnight at 4 °C in mouse anti-Cav-1 (1:50, BD Biosciences, Franklin Lakes, NJ, USA, Catalog# BD 610407) and rabbit anti-SNAIL (1:50, Santa Cruz Biotechnology Inc., Dallas, TX, USA, Catalog # sc-28199) primary antibodies. After washing in PBS, the cells were co-incubated in goat anti-mouse IgG (1:100, Life Technologies, Catalog # A-11001) and goat anti-rabbit IgG (1:100, Life Technologies Catalog # A1011) secondary antibodies for 1 h at room temperature. Cells were then incubated with DAPI (1:1000) for 20 min followed by washing in PBS. The chamber slides were mounted with antifade mounting media and imaged in a confocal microscope (Olympus FV1000). The intensity of Cav-1 and SNAIL were determined by using Image J software. Increased expression of both Cav-1 and SNAIL was observed in Cav-1-over H157 cells compared to mock cells (**P < 0.005) [file 12935_2019_892_MOESM4_ESM.jpg]
